# Degradation of Phosphonate Antiscalants in Reverse Osmosis Concentrate through Oxygenation of Fe(II)-Bearing Biotite

**DOI:** 10.1021/acsestengg.6c00154

**Published:** 2026-05-15

**Authors:** Lai Wei, Yifan Ding, Wencong Xing, Huize Xue, Omowunmi Sadik, Wen Zhang, Lijie Zhang

**Affiliations:** 1 Department of Chemistry and Environmental Science, 5965 New Jersey Institute of Technology, Newark, New Jersey 07102, United States; 2 Department of Civil and Environmental Engineering, 5965 New Jersey Institute of Technology, Newark, New Jersey 07102, United States

**Keywords:** phosphonate, Fe(II)-bearing mineral, reverse osmosis concentrate, hydroxyl radical, high-valent Fe species

## Abstract

Phosphonates are widely used in reverse osmosis (RO) and other filtration processes, making their removal critical for effective management of RO concentrate. While oxygenation of Fe­(II)-bearing minerals can generate powerful oxidants, such as hydroxyl radical (•OH) and high-valent Fe species, their efficacy in degrading phosphonates under the complex water chemistry of RO concentrate remains unclear. Here, we investigated the degradation of two representative phosphonates—nitrilotri­(methylphosphonic acid) (NTMP) and iminodi­(methylphosphonic acid) (IDMP)—using oxygenation of Fe­(II)-bearing biotite. NTMP degraded rapidly in the presence of biotite under oxic conditions, while IDMP showed negligible degradation. Mechanistic analyses indicate that both •OH and high-valent Fe species contributed substantially to NTMP degradation, driven by the oxygenation of biotite structural Fe­(II). Despite the complex composition of RO concentrate, NTMP degradation remained relatively consistent in the presence of biotite under neutral pH, although with slower kinetics. Moreover, the addition of acetate and oxalate accelerated NTMP degradation, even in RO concentrate. Overall, these findings reveal a mineral-mediated, sustainable Fenton-like pathway for phosphonate degradation and highlight the potential of Fe­(II)-bearing minerals as a greener strategy for managing phosphonates in RO concentrate, thereby reducing associated environmental impacts.

## Graphical abstract

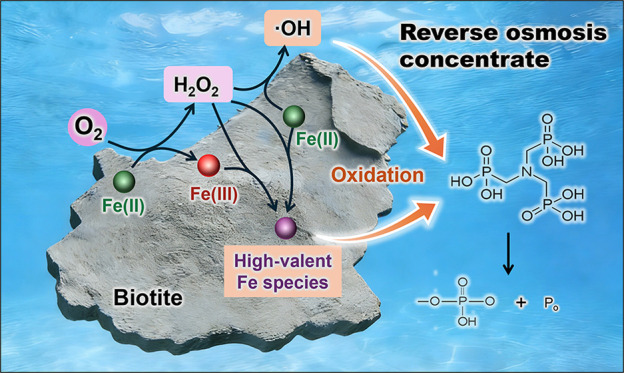

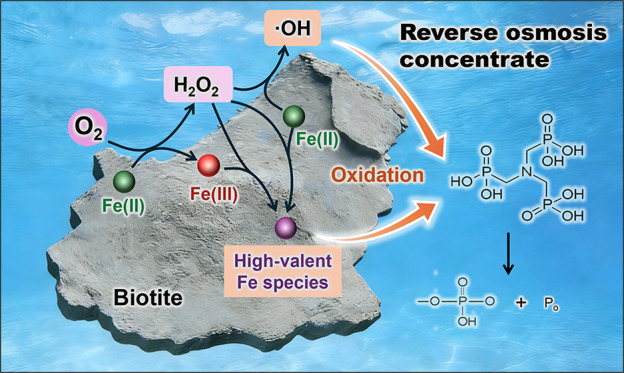

## 1 Introduction

Phosphonates, synthetic complexing agents containing one or more −PO­(OH)_2_ groups, are widely used as chelating agents and scale inhibitors in various industrial and domestic applications, such as water treatment, oil production, and household detergents.^1^ For instance, phosphonates are commonly used as antiscalants to prevent membrane scaling in reverse osmosis (RO) and other filtration processes. To address global freshwater shortage, membrane processes are increasingly employed in water reuse and desalination that ultimately generates large volume of waste streams such as RO concentrate.^2–5^ Moreover, the increasing osmotic pressure and membrane fouling cause RO membrane filtration to reject up to 20% of the influent as concentrate,^6^ which contains enriched levels of feedwater constituents, including added chemicals such as phosphonate antiscalants.^6^ The presence of phosphonates complicates RO concentrate management and disposal, as insufficient removal hinders the recovery of valuable minerals and salts through precipitation and crystallization as well as poses significant environmental threats,^1,5,6^ including eutrophication^7,8^ and ecotoxicological impacts.^9^ Thus, it is essential to implement appropriate removal technologies for phosphonates in RO concentrate.

Phosphonate removal from RO concentrate presents multifaceted challenges. For instance, phosphonates are resistant to biological degradation due to their stable C–P bonds, resulting in slow and incomplete biodegradation during conventional wastewater biological treatment processes.^1^ In addition, traditional physicochemical methods, such as ferric salt precipitation and flocculation, are often ineffective because phosphonates inhibit floc formation and generate excessive sludge volumes.^10^ Advanced oxidation processes (AOPs), such as ozonation, Fenton processes, UV/chlorine, and UV/persulfate, have demonstrated effectiveness in phosphonate degradation.^8,11^ For instance, UV/persulfate achieved up to 80% conversion of phosphonates to orthophosphate in deionized (DI) water at neutral pH,^8^ while UV/Fe^2+^/H_2_O_2_ enabled 50–80% phosphonate degradation via radical-induced cleavage of C–N and C–P bonds under similar pH conditions.^14^ Despite their effectiveness, AOPs raise several concerns. In waters containing halide ions, AOPs can generate toxic halogenated byproducts, such as trihalomethanes and haloacetic acids, posing environmental and health risks.^12^ Fe­(II)-based AOPs may produce substantial iron sludge that increases disposal costs and the risk of secondary pollution.^12^ Furthermore, the high concentrations of ionic species and organic matter contents in wastewater can quench reactive radicals and reduce degradation efficiency.^13^ For example, chloride ions could inhibit sulfate radical-based AOPs, leading to about 70% reduction in the apparent phosphonate degradation rate in RO concentrate compared to DI water during the UV/persulfate process.^8^ Beyond these technical challenges, AOPs are considered cost-intensive, due to their high energy and chemical demands.^14^ Hence, there is growing interest in alternative oxidation strategies that can achieve efficient contaminant removal in RO concentrate while minimizing energy input and secondary pollution.

Given the limitations of conventional AOPs, increasing attention has been directed toward alternative pathways for generating reactive oxygen species (ROS), including superoxide (O_2_•^–^) and hydroxyl radical (•OH). While •OH is a potent, nonselective oxidant effective against a wide spectrum of organic pollutants, O_2_•^–^ can contribute via selective oxidation or reductive transformation.^15^ Of particular interest are reactions between dissolved O_2_ and Fe­(II)-bearing minerals (e.g., reduced nontronite and pyrite),^16,17^ which can produce ROS. Ligands such as sodium tripolyphosphate (TPP) and ethylenediaminetetraacetic acid (EDTA) can further enhance ROS generation from Fe­(II)-bearing minerals by six to seven times, likely through increased electron utilization efficiency.^17^ Importantly, oxygenation of Fe­(II)-bearing minerals has been demonstrated to degrade various organic compounds in an aqueous environment under ambient conditions, including trichloroethene, 1,4-dioxane, and methylmercury.^18,19^ Phosphonate degradation in the presence of biotite, an Fe­(II)-bearing mica mineral, has also been reported under harsher conditions (95 °C, pH ∼ 3).^18^ Recent studies further identified high-valent Fe species [e.g., Fe­(IV)], which are strong and selective oxidants, during Fe­(II)-mineral oxygenation.^20,21^ Surface-generated Fe­(IV) can compete with •OH formation, which may explain the relatively low •OH yields observed in some systems.^20,21^ Notably, these high-valent Fe species can also degrade phosphonates,^22^ suggesting multiple potential pathways for phosphonate removal in oxygenated Fe­(II)-mineral systems.^20,21^ However, it remains unclear whether phosphonates can be degraded in real RO concentrate, which has complex water chemistries such as high ionic strength, diverse coexisting ions, and dissolved organic matter (DOM).

In this study, the kinetics and mechanisms of phosphonate degradation by Fe­(II)-bearing minerals under oxic conditions were investigated. Biotite was chosen as a representative Fe­(II)-bearing mineral due to its environmental abundance and high structural Fe­(II) content (∼ 40 wt %). Nitrilotris­(methylenephosphonic acid) (NTMP) was selected as a representative phosphonate because it is one of the most widely used nitrogen-containing phosphonate antiscalants in RO systems.^1,8^ Iminodi­(methylenephosphonic acid) (IDMP), a common degradation intermediate of NTMP that differs only by having two rather than three phosphonic groups,^1,8^ was additionally included to evaluate the extent of NTMP transformation and to improve the broader relevance of this study to other structurally related N-containing phosphonate antiscalants. The impacts of water chemistry relevant to RO concentrate on phosphonate degradation were evaluated, and the potential of various short-chain organic ligands to improve degradation was explored. Finally, the feasibility of applying this approach to degrade phosphonates in real RO concentrate was assessed.

## 2 Materials and Methods

### 2.1 Chemicals and Minerals

Chemicals, including monobasic potassium phosphate (KH_2_PO_4_, purity > 99%), IDMP and NTMP (purity > 95%), methyl phenyl sulfoxide (PMSO, > 97%), dimethyl sulfoxide (DMSO, > 99.9%), *tert*-butyl alcohol (TBA, > 99.5%), chloroform (> 99.8%), terephthalic acid (TA), EDTA, sodium acetate, trisodium citrate, and sodium oxalate (purity > 99%), were purchased from Sigma-Aldrich. 2,3-Bis­(2-methoxy-4-nitro-5-sulfophenyl)-2*H*-tetrazolium-5-carboxanilide (XTT) was purchased from Biotium. Suwannee River Humic Acid (HA) Standard III (product number: 3S101H) was purchased from International Humic Substances Society (IHSS), USA. HA stock solution was prepared by dissolving HA powder and adjusting the pH to 5 – 6 using 1 M NaOH and then filtered through a 0.45 μm nylon filter.^23^

Mica mineral biotite was purchased from Ward’s Natural Science (Rochester, New York) and was originally sourced from Bancroft, Ontario, Canada. Biotite features a fundamental 2:1 layer structure consisting of two opposing tetrahedral (T) sheets, with an intervening octahedral (O) sheet. The tetrahedral sheets contain Si and/or Al, while the octahedral sheet is occupied by Al, Mg, and/or Fe, with the overall charge of the TOT layer being balanced by interlayer cations (typically Na^+^ and K^+^).^24^ For pretreatment, Biotite was sequentially washed with ethanol, acetone, isopropanol, and deionized (DI) water (resistance > 18.2 MΩ·cm, EMD Millipore Direct-Q 3 system, Darmstadt, Germany) to ensure thorough removal of surface-adsorbed organic matters.^18,25^ These solvents were selected for their broad solubilizing capacity and minimal impact on the structure of biotite.^26^ After pretreatment, biotite was dried, ground, and sieved to obtain mineral particles sized between 53 and 106 μm.

### 2.2 Mineral Characterization

Variations in the oxidation states of Fe within the biotite before and after reactions were comparatively assessed using X-ray photoelectron spectroscopy (XPS), performed on a Thermo K-Alpha X-ray photoeletron spectrometer (Thermo Fisher Scientific, USA) employing a monochromated Al Kα X-ray source (*h*ν = 1486.6 eV). The C­(1s) level (284.8 eV) was taken as the reference binding energy. High-resolution spectra of Fe 2p were collected and fitted using a least-squares procedure with a Gaussian–Lorentzian peak sharp after subtracting a Smart baseline (Advantage 7.0).

The reduction of O_2_ on the biotite surface was investigated using cyclic voltammetry (CV) with a modified carbon paste electrode method.^27^ CV experiments were performed at room temperature (22 °C) using an Autolab PGSTAT302N potentiostat controlled by NOVA software (Version 2.1, Metrohm BV, Netherlands). Two types of carbon paste electrodes (CPEs) were prepared: (1) a biotite–graphite composite electrode composed of 60% graphite powder, 30% biotite, and 10% silicon oil as a binder, and (2) a graphite-only electrode composed of 70% graphite powder and 30% silicon oil. The compounds were manually homogenized using an agate mortar and pestle and then packed into Teflon sleeves and cured at room temperature for 24 h. CV measurements were conducted under three conditions: graphite-only electrode, biotite–graphite electrode with N_2_ purging for 30 min to remove dissolved O_2_ (anoxic), and biotite–graphite electrode without purging (oxic). A platinum wire (Metrohm–Autolab 6.0301.100) served as the counter electrode, and an Ag/AgCl electrode (Metrohm–Autolab 6.0733.100) was used as the reference electrode. During CV measurements, the potential was swept from −0.3 to 0.7 V at a scan rate of 10 mV·s^–1^ to better resolve redox peaks. The electrolyte solution was 0.1 M KCl (pH 6.5), which provided sufficient ionic conductivity without introducing additional redox-active species.

Fourier Transform infrared spectroscopy (FTIR) spectra were collected using a Nicolet iS50 FTIR spectrometer (Thermo Fisher Scientific, USA) equipped with a cooled deuterated triglycine sulfate detector. Measurements were conducted in attenuated total reflectance mode using a ZnSe crystal as the internal reflection element. Spectra were recorded in the wavenumber region of 4000 – 550 cm^–1^ with a spectral resolution of 2 cm^–1^, and 45 scans were performed for each sample. Data acquisition and analysis were conducted using the OMNIC software package.

### 2.3 Batch Degradation Experiments

Degradation experiments were conducted in a 10 mM NaCl solution with a mineral loading of 1 g·L^–1^ and a phosphonate concentration of 0.1 mM, unless noted otherwise, in 50 mL centrifuge tubes at room temperature (22 ± 2 °C). Each tube was initially filled with 20 mL reaction suspension with dissolved oxygen (DO) at a concentration of ∼9 mg·L^–1^. The degradation of two phosphonates, i.e., IDMP and NTMP, was first investigated at unadjusted initial pH values of 4.2 and 3.5, respectively. The tubes were placed on a tube rotator (SilentShake, Crystal, McKinney, Texas, USA) at 80 rpm to facilitate thorough mixing and effective contact between the mineral particles and the solution. Degradation experiments were performed in triplicate and in the dark to avoid any potential photolysis. Control experiments were conducted with no minerals added. Samples were gathered at a series of predetermined time intervals (0–120 h) and then filtered using 0.22 μm PTFE membrane filters that are shown to have minimum adsorption of phosphonates or their degradation byproducts.^28^ The filtrate was quenched by TBA for •OH at a volume ratio of 1:1 immediately and then analyzed for orthophosphate, a potential product commonly used to indicate phosphonate degradation.^8^

The orthophosphate concentration in the filtrate was determined using a UV–visible spectrophotometer (Infinite M200 PRO, Tecan, USA) using the molybdenum blue colorimetric method (EPA method 365.1). To determine both the initial P concentrations and the P adsorption onto biotite, total phosphorus (TP) in the filtrate before and after degradation was measured by using a persulfate digestion method (EPA method 365.1). The amount of P adsorbed onto biotite was calculated by subtracting the postdegradation TP concentration from the initial TP concentration. Aqueous Fe^2+^ was monitored based on the 1,10-phenanthroline method using an UV–visible spectrophotometer (Infinite M200 PRO, Tecan, USA). Hydroxylamine hydrochloride was used as a reductant to convert all Fe^3+^ to Fe^2+^ in water; thus, aqueous total Fe was determined using the same method established in Fe^2+^ measurement. The aqueous Fe^3+^ was calculated by the subtraction of the aqueous Fe^2+^ from the aqueous total Fe.

### 2.4 Evaluation of the Roles of ROS and High-Valent Fe Species in Phosphonate Degradation

Based on previous studies,^16,20,29,30^ we hypothesized that ROS and high-valent Fe species, generated by biotite under oxic conditions could be responsible for phosphonate degradation. To test our hypothesis and qualitatively evaluate the roles of ROS in phosphonate degradation, TBA was employed as a selective •OH scavenger without influencing high-valent iron species [*k*_(•OH, TBA)_ = 6.0 × 10^8^ M^–1^ s^–1^] and chloroform was used to quench O_2_•^–^ [*k*_(O2•_–_, chloroform)_ = 3.0 × 10^10^ M^–1^ s^–1^].^21,31,32^ DMSO and PMSO can be oxidized by both •OH and high-valent Fe species; therefore, both compounds were employed as scavengers for •OH and high-valent Fe species.^20,33^ NTMP degradation in the presence of the respective scavengers was conducted and compared with experiments performed without scavengers.

### 2.5 Phosphonate Degradation Assessment under Varied Water Chemistry Conditions and in Real RO Concentrate

The degradation of NTMP in the presence of biotite was further evaluated under various water chemistry parameters relevant to RO concentrate.^34^ Specifically, the pH of untreated RO concentrate is generally reported to range from approximately 6.2 to 8.2,^34^ while downstream treatment processes may shift the pH to either acidic or basic conditions depending on the methods used.^35,36^ Therefore, NTMP degradation was investigated at pH 3.5, 7, and 9. The effects of ionic strength in the range of 10–1400 mM as NaCl were also examined. Additionally, the impacts of coexisting ions typically found in RO concentrate were assessed, including anions such as bromide (Br^–^), nitrate (NO_3_^–^), sulfate (SO_4_^2–^), and bicarbonate (HCO_3_^–^) at concentrations of 2, 10, 100, and 50 mM, respectively. The influence of common cations, including 70 mM calcium (Ca^2+^), 20 mM potassium (K^+^), and 300 mM magnesium (Mg^2+^), were also examined. Furthermore, the effects of HA on NTMP degradation were explored at concentrations of 4.6 and 2.3 mg-C·L^–1^. The effects of individual organic ligands (0.1 mM), including EDTA, acetate, citrate, and oxalate, on degradation of 0.1 mM NTMP at pH 7 were also examined. Moreover, to promote practical application potential, organic ligands showing enhancement on phosphonate will be tested in the presence of coexisting ions and HA that inhibited phosphonate degradation.

Additional experiments were performed in real RO concentrate obtained from Yuma Desalination Plant, Arizona, USA, which was predetermined to contain 0.04 mM orthophosphate and 0.07 mM organic P (see Table S1 for detailed characterization). To evaluate the degradation of NTMP under realistic water chemistry conditions, an additional 0.1 mM NTMP was spiked into the real RO concentrate.

### 2.6 Data Analysis

All the degradation experiments were run in triplicates. Data points in all figures represent the average of four to six replicate samples (at least two batch experiments), with error bars indicating one standard deviation. Significance of differences in the released orthophosphate at equilibrium during NTMP degradation among different test groups was evaluated by analysis of variance (ANOVA) in conjunction with Tukey’s HSD test using a significance level of 0.05 (Table S2).

## 3 Results and Discussion

### 3.1 Phosphonate Degradation in the Presence of Biotite under Oxic Conditions

The degradation kinetics of NTMP and IDMP were indicated by the release of orthophosphate, which is a common product from phosphonate degradation.^8,11,37^ The adsorption capacities of biotite toward orthophosphate, IDMP, and NTMP were evaluated first and found to be negligible (Figure S1), suggesting that adsorption had minimal impact on their aqueous concentrations. As shown in Figure 1a, the mineral-free control showed negligible NTMP degradation throughout the experiment, indicated by near-zero orthophosphate concentrations over time. In contrast, significant NTMP degradation was observed in the presence of biotite under oxic conditions, with the orthophosphate concentration rapidly increased to approximately 0.1 mM within the first 24 h and plateaued at around 0.12 mM after 48 h (Figure 1a), corresponding to ∼ 40% of the total P originally present in 0.1 mM NTMP. Previous studies suggest that NTMP degradation typically proceeds through initial C–P and C–N cleavage, with each NTMP molecule yielding one orthophosphate molecule and one IDMP as a key product,^8^ owing to its greater stability compared to NTMP. This enhanced stability likely arises from its simpler molecular structure (Figure 1), in which the amine group bonded to fewer reactive phosphonate moieties, rendering IDMP less susceptible to oxidative degradation.^38^ Consistently, negligible IDMP degradation was observed under the same experimental conditions (Figure 1b); therefore, we speculated that it accumulated as a stable intermediate during NTMP degradation.^8,37^ Overall, these results indicate that nearly all initially added NTMP was transformed, with at least one phosphate released per molecule.

**Figure 1:**
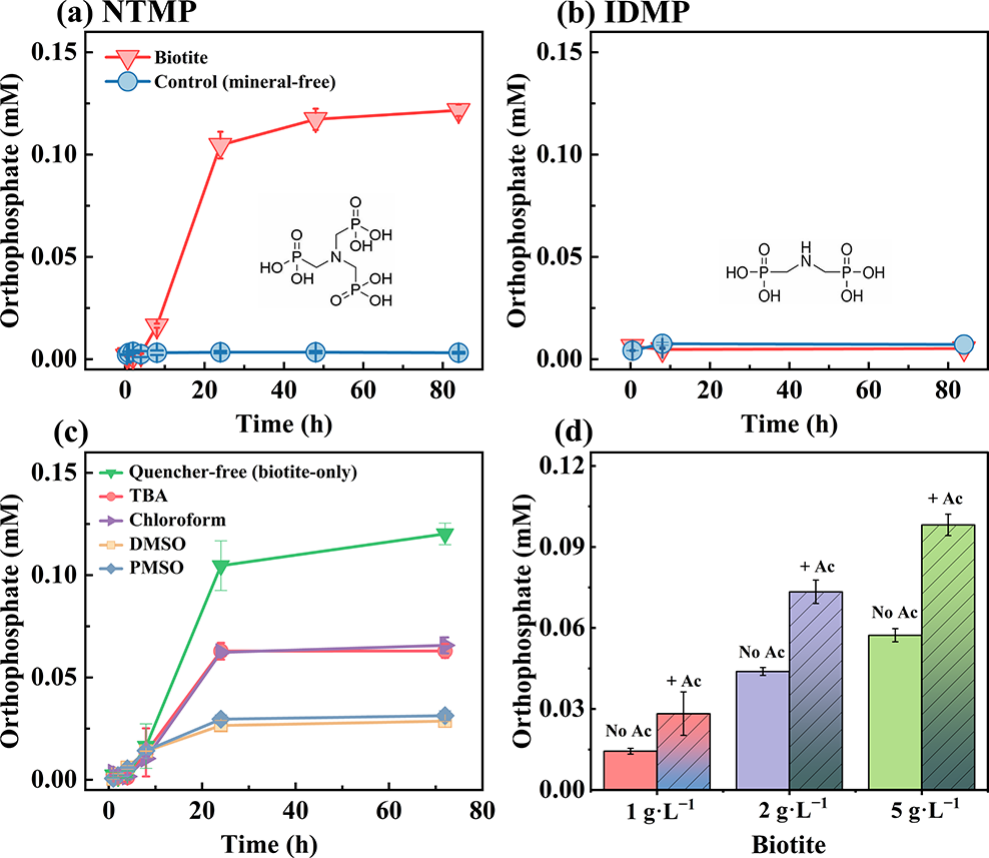
Kinetics of orthophosphate release from degradation of 0.1 mM (a) NTMP and (b) IDMP without (mineral-free control) and with the presence of biotite at respective initial pH of 2.9 and 3.5 under oxic conditions. (c) Kinetics of orthophosphate release from degradation of 0.1 mM NTMP with biotite at pH of 3.5 in the presence of 100 mM each scavenger. (d) Effects of acetate (Ac) addition (0.1 mM) on orthophosphate release after 24 h of NTMP (0.1 mM) degradation in the presence of 1, 2, and 5 g·L^–1^ biotite in real RO concentrate.

### 3.2 Roles of ROS and High-Valent Fe Species in Phosphonate Degradation

To understand the mechanisms and explore the roles of key oxidants via biotite oxygenation in phosphonate degradation, TBA and chloroform were used as scavengers for •OH and O_2_•^–^,^31^ respectively (Figure 1c). When TBA was present, equilibrium orthophosphate release decreased by approximately 47.5% compared to the quencher-free system, indicating significant inhibition and suggesting that •OH as a primary oxidant for NTMP degradation. This observation is consistent with previous studies reporting that •OH can be produced during the oxygenation of Fe­(II)-bearing minerals.^16,17,19^ In coupled mineral-Fe­(II) and O_2_ systems, •OH may be generated via two pathways: (i) single-electron transfer that sequentially produces O_2_•^–^ and H_2_O_2_, or (ii) two-electron transfer to O_2_ that directly forms H_2_O_2_.^29^ In both pathways, H_2_O_2_ further reacts with mineral-Fe­(II) through Fenton-like reaction to generate •OH.^16,19^ Experiments using chloroform as a selective quencher for O_2_•^–^ showed inhibition levels comparable to those observed with TBA during NTMP degradation, indicating that O_2_•^–^ was not directly involved in NTMP degradation but instead served as precursor to •OH formation. Quenching O_2_•^–^ with chloroform suppressed •OH generation, thereby inhibiting phosphonate degradation. These results suggest that ROS generation in the biotite oxygenation system was predominantly governed by a single-electron transfer pathway (Figure 2e), which is kinetically more favorable than the two-electron transfer pathway, as reported in previous studies.

**Figure 2:**
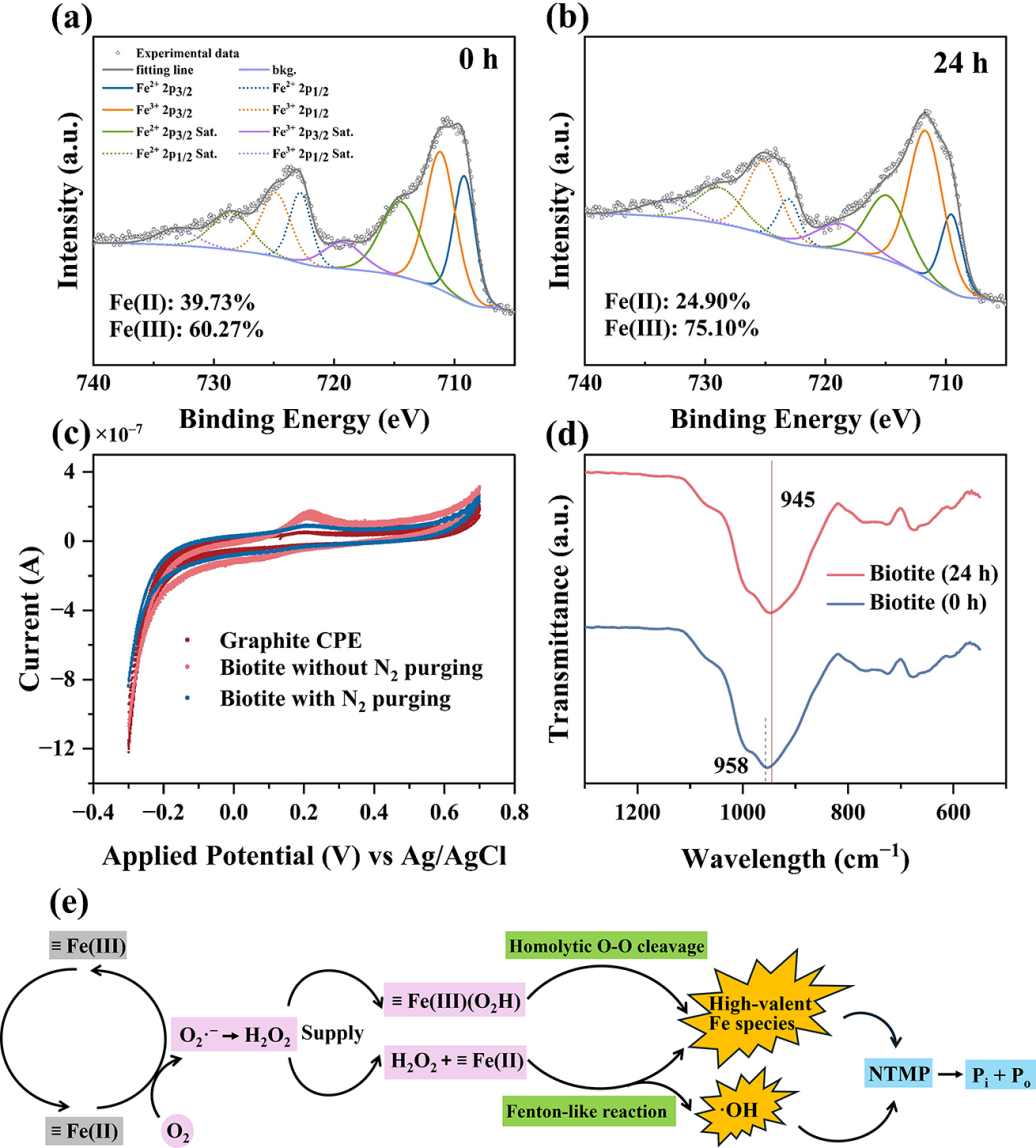
(a, b) XPS spectra of the biotite before (0 h) and after (24 h) reaction. Areas under blue peaks refer to the contribution of Fe­(II), and those under orange refer to the contributions of Fe­(III) speciation.^40^ The binding energy and full width at half-maximum (fwhm) of Fe­(2p) are shown in Table S3. (c) Cyclic voltammograms of graphite CPE and biotite-graphite electrodes with and without N_2_ purging in 0.1 M KCl, recorded at a scan rate of 10 mV·s^–1^ with applied potential swept from −0.3 to 0.7 V. Ag/AgCl was used as the reference electrode. (d) FTIR-ATR spectra of the before (0 h) and after (24 h) oxidation. (e) Schematic diagram of Fe­(II) oxidation generating oxidants for NTMP degradation.

Moreover, the partial inhibition of NTMP degradation by the •OH scavenger implies that other oxidants, such as high-valent Fe species that have been reported to form during the oxygenation of Fe­(II)-bearing minerals, may also contribute.^20,21^ DMSO and PMSO were thus employed as scavengers for both •OH and high-valent Fe species. The relative contribution of high-valent Fe species to NTMP degradation was determined by comparing their inhibitory effects with those of TBA. Specifically, the addition of DMSO/PMSO inhibited approximately 80.8% of orthophosphate release from NTMP degradation, indicating that high-valent Fe species accounted for ∼ 33.3% of overall NTMP degradation. It has been reported that the generation of •OH and high-valent Fe species [e.g., Fe­(IV)] proceeds via two competing pathways (Figure 2e): (i) stepwise O_2_ reduction to O_2_•^–^/H_2_O_2_ followed by •OH generation and (ii) formation of the precursor H_2_O_2_–Fe­(III) complex [≡Fe­(III)­(O_2_H)] at mineral surface sites, where homolytic cleavage of the O–O bond yields structural Fe­(IV), or Fe­(IV) can be generated by the reaction between Fe­(II) and H_2_O_2_.^20,30,39^ Together, the findings suggest that both •OH and high-valent Fe species serve as the major oxidants responsible for NTMP degradation in the biotite-O_2_ system.

### 3.3 Characterizations of Biotite Oxidation under Oxic Conditions

#### 3.3.1 Chemical State Analysis

To further elucidate the transformation of Fe­(II) in biotite during NTMP degradation and to confirm its oxidation in the formation of ROS, an XPS analysis was conducted. The Fe 2p3/2 peak was deconvoluted into Fe­(II) and Fe­(III) components, with binding energies of approximately 709.6 eV for Fe­(II) (FeO) and 710.8 eV for Fe­(III) (Fe_2_O_3_), respectively.^40^ Initially at 0 h (Figure 2a), Fe­(II) constituted approximately 39.7% of the total Fe in biotite, with Fe­(III) making up the remaining 60.3%. After 24 h of reaction (Figure 2b), the Fe­(II) content decreased to 24.9%, while Fe­(III) increased correspondingly to 75.1%. The substantial decline of Fe­(II) content on the biotite surface indicated that structural Fe­(II) near the mineral surface participates actively in redox processes. This result suggests that surface Fe­(II) is progressively oxidized during NTMP degradation, while the remaining interior Fe­(II) may continue to supply electrons through Fe­(II)–O–Fe­(III) transfer within the mineral lattice.^41^ Therefore, Fe in biotite likely undergoes partial Fe­(II)/Fe­(III) cycling during the reaction, which can sustain ROS generation for a certain period but gradually weaken as reactive Fe­(II) is depleted. Moreover, the dissolved Fe^2+^ and Fe^3+^ in the filtrate were nondetectable, suggesting that Fe­(II) oxidation predominantly occurred on the mineral surface rather than in solution after dissolution.

#### 3.3.2 Analysis of Electron Transfer in Fe­(II) Oxidation

Cyclic voltammetry was employed to investigate the electron transfer processes involved in biotite structural Fe­(II) oxidation and to gain insights into the corresponding reaction pathway. Figure 2c shows the cyclic voltammograms of graphite and biotite-graphite paste electrodes under oxic and anoxic conditions (purged with N_2_ to remove dissolved O_2_). In the oxygen-rich electrolyte, the biotite-graphite electrode displayed a distinct anodic peak around +0.2 V (vs Ag/AgCl), while no clear redox features were observed in the oxygen-depleted electrolyte or with the pure graphite electrode. This peak may be attributed to the oxidation of Fe­(II) to Fe­(III) within the biotite structure. Although the standard redox potential of the Fe­(III)/Fe­(II) couple is approximately +0.56 V vs Ag/AgCl, previous studies have shown that structural Fe­(II) of silicate minerals often exhibits lower redox potentials (0.13 – 0.32 V vs Ag/AgCl), due to lattice constraints and structural coordination effects.^42,43^ The shift in the redox potential of structural Fe­(II) makes it a better electron donor to react with dissolved O_2_ and produce ROS. Moreover, these effects allows structural Fe­(II) to react with O_2_ at rates several orders of magnitude faster than dissolved Fe­(II).^42^ Hence, the observed oxidation peak suggests that oxygen reduction on Fe­(II)-bearing biotite is a surface-mediated process. The absence of a cathodic peak indicates that the product of the oxidation is either unstable or has undergone a secondary chemical change. Consistently, scan-rate-dependent CV (Figure S4) reveals that the cathodic feature becomes increasingly discernible with higher scan rates yet remains substantially less prominent than the anodic peak. This behavior is consistent with an EC-type mechanism, wherein the oxidized Fe­(III) intermediate undergoes rapid chemical transformation, e.g., the formation of high-valent Fe species, thereby limiting full electrochemical reversibility.

#### 3.3.3 Surface Functional Group Assessment

Figure 2d presents the ATR-FTIR spectra of biotite before and after 24 h of reaction with NTMP. Both spectra show the characteristic Si–O stretching vibrations between ∼ 900 and 1100 cm^–1^, consistent with the typical structural features of trioctahedral micas.^44^ Specifically, the Si–O stretch appears near 958 cm^–1^ at 0 h but shifts slightly to ∼ 945 cm^–1^ after reaction. This shift can be attributed to changes in the local lattice environment, likely reflecting partial conversion of Si–O–Fe­(II) to Si–O–Fe­(III) bonds as Fe­(II) is oxidized within the octahedral layers.^41,44^ The band between 3800 and 3600 cm^–1^ is typically assigned to OH stretching, reflecting the presence of MgFe^3+^OH and/or AlFe^2+^OH groups, or mixed cationic sites in the octahedral layer.^44^ Moreover, no additional bands appear between 1400 and 400 cm^–1^, suggesting that the fundamental local bonding and coordination environment of biotite remained unchanged over the 24 h reaction. Instead, the observed band change pointed to minor coordination adjustments as structural Fe­(II) oxidation altered the local bonding environment.

Collectively, XPS, cyclic voltammetry, and FTIR results consistently indicated that the oxidation process observed is dependent on both structural Fe­(II) and dissolved O_2_, with O_2_ acting as the primary electron acceptor. The observed shift in Fe speciation highlights that structural Fe­(II) plays a crucial role as an electron donor during NTMP degradation, likely through the generation of •OH and high-valent Fe species, consistent with previous studies.^16,45,46^

### 3.4 Effects of Aqueous Chemistry on NTMP Degradation

#### 3.4.1 pH

Figure 3a shows that under both acidic (pH 3.5) and neutral (pH 7) conditions, NTMP degradation by biotite under oxic conditions was rapid and comparable, reaching an equilibrium orthophosphate concentration of approximately 0.12 mM after 24 h. In comparison, at weak alkaline conditions (pH 9), approximately 31.2% lower orthophosphate was observed compared to neutral and acidic conditions, suggesting inhibited NTMP degradation. A previous study reported that the second-order rate constant of •OH with NTMP did not change significantly across a pH range of 4 to 11.5 [*k*_(•OH, NTMP)_ = (1.1 ± 0.1) × 10^8^ M^–1^ s^–1^].^8^ It is likely that the high pH of 9 suppressed •OH yield, as the transformation of O_2_•^–^ into H_2_O_2_ is less favorable under alkaline conditions.^30,31^ Simultaneously, H_2_O_2_ dissociates into HO_2_^–^, which reacts with •OH to form the less reactive HO_2_^–^, thus reducing degradation efficiency. Moreover, the nonradical decay of H_2_O_2_ at high pH would also reduce the production of high-valent Fe species, leading to decreased NTMP degradation.^30^ Alternatively, the degradation efficiency at different pH values could be influenced by changes in NTMP speciation. NTMP has six p*K*a values of 0.3, 1.5, 4.6, 5.9, 7.3, and 12.1,^47^ and its dominant ionic species become increasingly negatively charged as pH increases. Concurrently, the biotite surface also becomes more negatively charged as pH increases (Figure S2), leading to stronger electrostatic repulsion between the mineral surface and negatively charged P species, thereby inhibiting NTMP degradation.^22^

**Figure 3:**
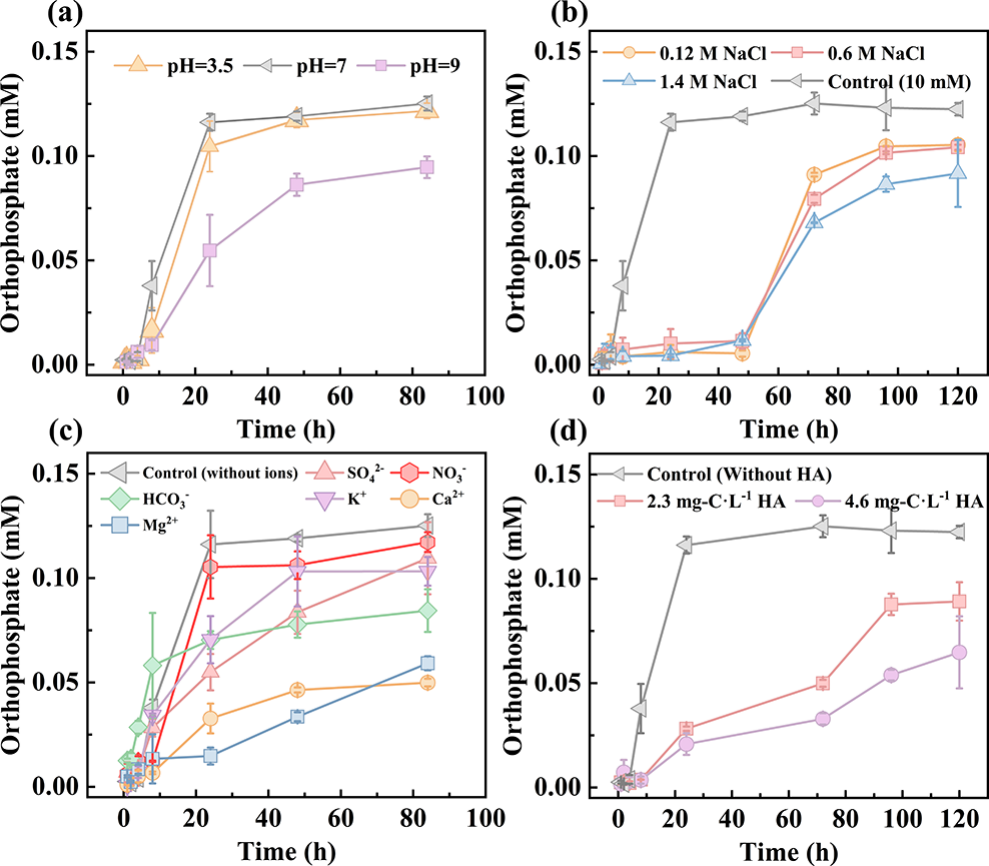
Degradation kinetics of 0.1 mM NTMP in 10 mM NaCl solution by oxidation of 1 g·L^–1^ biotite. (a) Effects of pH on NTMP degradation. (b – d) Influence of (b) varying NaCl concentrations, (c) different coexisting ions, and (d) various concentrations of HA at pH 7. A summary of Tukey HSD *p*-value comparisons for the effects of pH, ionic strength, coexisting ions, and HA on orthophosphate release at equilibrium during NTMP degradation is included in Table S2.

#### 3.4.2 Salinity and Coexisting Ions

Given the high salinity and complex coexisting ions typically found in RO concentrate, the effects of ionic strength on phosphonate degradation in the biotite-O_2_ system were explored using NaCl solutions at concentrations representative of brackish water (0.12 M), seawater (0.6 M), and RO concentrate (1.4 M).^34^ The results indicate that high concentrations of NaCl significantly slowed down the degradation of NTMP (Figure 3b). Compared with the control (10 mM NaCl), where NTMP degradation reached equilibrium after 24 h with ∼0.12 mM orthophosphate generated, orthophosphate was released only after 48 h and stabilized at 0.09–0.10 mM after 96 h under higher salinity conditions. This inhibition can primarily be attributed to the scavenging of •OH by Cl^–^, which forms less reactive species such as Cl•, Cl_2_•^–^, or ClOH•^–^ [*k*_(•OH, Cl)_ = 4.3 × 10^9^ M^–1^ s^–1^, *k*_(ClOH•_^–^_, H_^+^_)_ = 2.1 × 10^10^ M^–1^ s^–1^, *k*_(Cl•,,Cl_^–^_)_ = 8.5 × 10^9^ M^–1^ s^–1^].^8,48^ In contrast, previous studies have shown that high-valent Fe species were much less affected by Cl^–^ at pH ≥ 3.^49,50^ Therefore, under high-salinity conditions, NTMP degradation is mainly inhibited by •OH scavenging by Cl^–^, rather than by scavenging of high-valent Fe species.

The performance of biotite in degrading NTMP was further investigated in the presence of various coexisting ions (Figure 3c). The presence of NO_3_^–^, K^+^, and SO_4_^2–^ did not significantly affect the degradation of NTMP. In contrast, in the presence of HCO_3_^–^, Ca^2+^, and Mg^2+^ ions, the detected equilibrium orthophosphate concentrations were only ∼56% of those observed with the other ions. The inhibition by HCO_3_^–^ may be attributed to the reaction between •OH and HCO_3_^–^, forming less reactive HCO_3_^•–^ species, which can hardly degrade NTMP [*k*_(HCO3_^–^_, •OH)_ = 8.5 × 10^6^ M^–1^ s^–1^].^51–53^ Moreover, CO_3_^2–^ or HCO_3_^–^ can form a surface complex with Fe­(II) of biotite, thereby reducing active sites for both •OH and high-valent Fe species.^53^ For Ca^2+^ and Mg^2+^, prior studies report that their complexation with NTMP can alter the electron density around the nitrogen center, rendering the C–N bond more vulnerable to nucleophilic attack and thereby enhancing •OH-mediated degradation.^54^ However, because the detected orthophosphate concentrations reflect the net effect of degradation and secondary precipitation, the lower observed orthophosphate levels in the presence of high Ca^2+^ and Mg^2+^ concentrations may be attributed to the precipitation of insoluble calcium or magnesium phosphate minerals. Thermodynamic calculations using Visual MINTEQ indicated that, under the experimental conditions (0.12 mM orthophosphate, 10 mM NaCl, and 70 mM Ca^2+^, or 300 mM Mg^2+^ at pH 7), the system was supersaturated with respect to several calcium phosphate phases, including β-Ca_3_(PO_4_)_2_ and hydroxyapatite, whereas Mg phosphate phases remained slightly undersaturated. Although homogeneous Mg phosphate precipitation is thermodynamically unfavorable, heterogeneous precipitation on the biotite surface cannot be excluded because mineral surfaces may locally promote nucleation and precipitation due to a lower energy barrier.^55,56^ It has also been reported that the intrinsic antiprecipitation capacity of NTMP decreases upon degradation,^13,57^ which would further promote the formation of phosphate precipitates.

#### 3.4.3 DOM

RO concentrate typically contains high levels of DOM, which is widely recognized as an important sequester for •OH.^22,45^ NTMP degradation in the presence of 2.3 and 4.6 mg-C·L^–1^ of HA showed that HA not only slowed down the kinetics but also reduced the extent of NTMP degradation at equilibrium, with stronger inhibition at the higher HA concentration (Figure 3d). In the absence of HA, NTMP degradation generally reached an equilibrium after 24 h, releasing about 0.12 mM orthophosphate. However, the presence of HA substantially extended the time required to reach equilibrium. Specifically, with 2.3 mg C·L^–1^ of HA, it took 120 h to produce about 0.065 mM orthophosphate, while with 4.6 mg C·L^–1^ HA, equilibrium was reached at 96 h, releasing approximately 0.088 mM orthophosphate. These results suggest that HA likely competed with NTMP for the produced •OH and high-valent Fe species, thereby influencing the rate and extent of NTMP degradation.^13,50^

### 3.5 Effects of Short-Chain Organic Ligands on NTMP Degradation

Recent studies have shown that the presence of organic ligands, such as EDTA, can significantly enhance the degradation of organic contaminants during Fe­(II)-bearing sediment oxygenation via enhancing electron transfer processes.^17,19^ Motivated by these findings, we investigated the effects of different organic ligands on NTMP degradation in the presence of biotite and evaluated whether ligand addition can alleviate the inhibitory effects of 1.4 M NaCl, 4.6 mg C·L^–1^ HA, 50 mM HCO_3_^–^, 70 mM Ca^2+^, or 300 mM Mg^2+^. Figure 4a shows that the addition of acetate and oxalate significantly accelerated NTMP degradation kinetics compared to the ligand-free control, leading to a rapid increase in orthophosphate concentration and achieving equilibrium within approximately 8 h. The enhancement observed with acetate and oxalate may stem from improved electron utilization efficiency of mineral-associated Fe­(II) for •OH generation, as reported by previous studies.^17,19^ In the absence of ligands, electrons released from structural Fe­(II) in minerals are gradually transferred to O_2_ through a stepwise mechanism. A recent study on the oxidation of reduced nontronite reported that edge Fe­(II) and trioctahedral Fe­(II)–Fe­(II)–Fe­(II) entities were rapidly oxidized, with electrons ejected directly to O_2_.^41^ At later stages, slower oxidation of interior Fe­(II) occurred as electrons migrate through Fe­(II)–O–Fe­(III) linkages to edge sites. This process sustained •OH production but was limited by slow electron transfer within the mineral structure. In contrast, the addition of certain short-chain organic ligands, e.g., acetate, can significantly alter this pathway by enhancing electron transfer.^17^ Other studies have also reported that short-chain organic acids can enhance the dissolution of minerals, such as reduced nontronite and pyrite, promoting the formation of aqueous ligand–Fe^2+^ complex.^17,58,59^ In this process, the homogeneous Fenton reaction, facilitated by the oxidation of aqueous ligand–Fe^2+^ complex, contributes to •OH generation.^17,58^ However, in the biotite system, homogeneous reactions involving an aqueous ligand–Fe^2+^ complex was likely limited because biotite is substantially less susceptible to ligand-promoted dissolution under ambient conditions. Notably, the addition of EDTA and citrate completely inhibited NTMP degradation, as indicated by the negligible changes in orthophosphate concentrations over the reaction period (Figure 4a). This result indicates that the inhibitory effect of the added EDTA and citrate outweighed any potential enhancement associated with ligand-promoted Fe dissolution. One likely explanation is that EDTA and citrate may primarily act as competing organic substrates for •OH, due to their higher reaction rate constants with •OH compared to NTMP [*k*_(•OH, EDTA)_ = 4.0 × 10^8^ M^–1^ s^–1^,^32^ *k*_(•OH, citrate)_ = 1.5 × 10^8^ M^–1^ s^–1^,^60^ and *k*_(•OH, NTMP)_ = (1.1 ± 0.1) × 10^8^ M^–1^ s^–1^].^8^ This observation is consistent with a previous study reporting that excess EDTA suppressed trichloroethylene degradation when present in excess.^17^ In addition, EDTA and citrate may also compete with NTMP for high-valent Fe species, thereby inhibiting NTMP degradation.^61^

**Figure 4:**
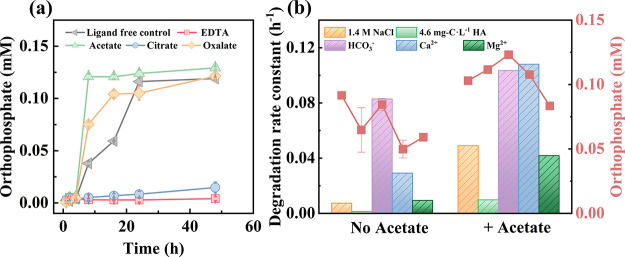
(a) Degradation kinetics of 0.1 mM NTMP in the presence of various organic ligands (0.1 mM). (b) Degradation rate constants of 0.1 mM NTMP and corresponding orthophosphate release at 24 h under various inhibitors (i.e., 1.4 M NaCl, 4.6 mg C·L^–1^, 50 mM HCO_3_^–^, 70 mM Ca^2+^, and 300 mM Mg^2+^), in the absence and presence of acetate. Other important experimental conditions: 1 g·L^–1^ biotite in 10 mM NaCl solution at pH of 7.

Figure 4b demonstrates the combined effects of acetate and the other aqueous inhibitors identified in Figure 3 on NTMP degradation. NTMP degradation kinetics was fitted using a pseudo-first-order kinetics model to calculate rate constants (*k*), as described in eq 1.^62^

$$q_{t}=q_{e}\left(1-e^{-kt}\right)$$ 1

where *t* (h) is the reaction time, *q*_t_ (mM) and *q*_e_ (mM) are the respective amounts of orthophosphate released from NTMP at any time *t* and at equilibrium, respectively, and *k* (h^–1^) is the rate constant of the pseudo-first-order model. Both *k* values and equilibrium orthophosphate concentrations were used to evaluate the effects of different inhibitors.

The addition of acetate significantly increased both the pseudo-first-order rate constant of orthophosphate release and its equilibrium concentration across different inhibitor conditions (Figure 4b and Figure S3). In the presence of HA, NaCl, and HCO_3_^–^, *k* increased from 0.00138 to 0.00985, 0.007 to 0.050, and 0.082 to 0.103 h^–1^, respectively. The corresponding orthophosphate concentrations at equilibrium rose from 0.065 to 0.112, 0.092 to 0.103, and 0.085 to 0.123 mM, respectively. Consistent with the previous observations, these enhancements are likely attributable to acetate-induced improvements in electron efficiency, which promote •OH production and thereby accelerate NTMP degradation and orthophosphate release. Furthermore, in systems containing Ca^2+^ and Mg^2+^, acetate addition increased *k* from 0.029 to 0.108 and from 0.010 to 0.042 h^–1^, respectively. The equilibrium orthophosphate concentrations also increased from 0.050 to 0.108 mM and 0.059 to 0.083 mM. These improvements may be due to the acetate-cation complexation that may reduce the formation of insoluble Ca- or Mg-phosphate precipitates and thus increase the measurable orthophosphate concentration.

### 3.6 Evaluation of NTMP Degradation in Real RO Concentrate

To evaluate the applicability of ligand-assisted biotite oxygenation for phosphonate degradation under realistic conditions, we tested the effects of biotite dosage and acetate in a real RO concentrate spiked with 0.1 mM NTMP. After 24 h, approximately 0.014 mM orthophosphate was released with 1 g·L^–1^ biotite alone, while acetate addition doubled the release to around 0.028 mM (Figure 1d). These results suggest that, although acetate enhanced orthophosphate release, the overall NTMP degradation remained limited, possibly due to •OH consumption by impurities present in the RO concentrate. Increasing the biotite dosage to 2 and 5 g·L^–1^ further elevated orthophosphate concentrations, yielding approximately 0.044 and 0.057 mM without acetate, and 0.073 and 0.092 mM with acetate, respectively. These results confirm that both mineral-Fe­(II) dosage and acetate addition enhance NTMP degradation in real RO concentrate, with the stronger effect of higher biotite dosage attributed to increased availability of structural Fe­(II) and greater •OH production. Previous studies have reported that, although NTMP is not completely degraded, its degradation products display significantly weakened antiprecipitation capabilities.^13,57^ Such transformations reduce the inhibitory effects of NTMP on mineral precipitation and salt crystallization during RO concentrate treatment and also enable subsequent orthophosphate adsorption and reuse. We also note that future work should focus on developing approaches that more effectively enhance oxidant production and utilization for phosphonate degradation.

## 4 Conclusions

This study demonstrates the potential of biotite-enhanced NTMP degradation as a pretreatment approach for RO concentrate management. Our results indicate that Fe­(II)-bearing biotite effectively degraded NTMP under oxic conditions, releasing orthophosphate and IDMP as major stable products, potentially diminishing its chelating capability and antiprecipitation properties, thereby facilitating subsequent mineral recovery through precipitation from RO concentrate. The degradation of NTMP was likely driven by •OH and high-valent Fe species, generated via the oxygenation of mineral structural Fe­(II). XPS and cyclic voltammetry analysis confirmed the oxidation of structural Fe­(II) in biotite, and FTIR spectra further indicated changes in local bonding environments consistent with Fe­(II) oxidation. NTMP degradation was influenced by pH, ionic strength, coexisting ions, and DOM, while acetate and oxalate enhanced degradation kinetics by promoting electron transfer and alleviated the inhibitory effects. In real RO concentrate, effective NTMP degradation was achieved with improved orthophosphate release by increasing biotite dosage and acetate addition. These results highlight the potential of biotite-based oxidation as a practical strategy for phosphonate removal from RO concentrate for its management.

## Supplementary Material



## Notes

Characteristics of reverse osmosis (RO) concentrate obtained from Yuma Desalination Plant, Arizona; summary of one-way ANOVA results for the effects of pH, ionic strength, coexisting ions, and HA on NTMP degradation; binding energy and full width at half-maximum (fwhm) of Fe­(2p) in biotite before (0 h) and after (24 h) oxidation characterized by XPS; adsorption kinetics of orthophosphate and NTMP by biotite (1 g·L^–1^) in 10 mM NaCl at pH 7; zeta potential of biotite particles at varied pH in 10 mM NaCl; orthophosphate release from degradation of 0.1 mM NTMP in the presence of acetate with various inhibitors (PDF)
