# Supplementary material for: Degradation of Phosphonate Antiscalants in Reverse Osmosis Concentrate through Oxygenation of Fe(II)-Bearing Biotite
Source: ACS ES T Eng. 2026 May 15;6(6):1839–48. doi: 10.1021/acsestengg.6c00154 (PMC13270527; doi:10.1021/acsestengg.6c00154)
Supplement: Supplementary file 1 [file ee6c00154_si_001.pdf]

Supporting Information for

Degradation of phosphonate antiscalants in reverse osmosis concentrate through oxidation of Fe(II)-bearing biotite

Lai Wei[1], Yifan Ding[1], Wencong Xing[1], Huize Xue[1], Omowunmi Sadik[1], Wen Zhang[2], Lijie Zhang[1,*]

[1] Department of Chemistry and Environmental Science, New Jersey Institute of Technology, Newark, NJ 07102, USA

[2] Department of Civil and Environmental Engineering, New Jersey Institute of Technology, Newark, NJ 07102, USA

***Corresponding author:** Lijie Zhang, lijie.zhang@njit.edu

Table S1. The characteristics of reverse osmosis (RO) concentrate obtained from Yuma Desalination Plant, Arizona.

Parameter	Concentration	Units
Conductivity	8,498.9±11.5	$\mu S \cdot cm^{-1}$
pH	6.5±0.1	(measured at 19.5 °C)
ORP	149±1	mV
Dissolved oxygen	8.3±0.1	$mg \cdot L^{-1}$
Total dissolved solids	5,990.5±6.8	$mg \cdot L^{-1}$
Turbidity	6.4±0.5	NTU
Nitrate as N	164.7	(~11.8 mM)
Aluminum	0.07	$mg \cdot L^{-1}$
Barium	0.04	$mg \cdot L^{-1}$
Bicarbonate as $CaCO_3$	11.3	$mg \cdot L^{-1}$
Boron	1.47	$mg \cdot L^{-1}$
Calcium as $CaCO_3$	775	$mg \cdot L^{-1}$
Chloride	1,920	$mg \cdot L^{-1}$
Chromium	0.15	$mg \cdot L^{-1}$
Fluoride	<20	$mg \cdot L^{-1}$
Iron	1.53	$mg \cdot L^{-1}$
Magnesium as $CaCO_3$	650	$mg \cdot L^{-1}$
P - PO_4^{3-}	1.21	$mg \cdot L^{-1}$
Total Phosphorus	3.41	$mg \cdot L^{-1}$
Potassium	32.1	$mg \cdot L^{-1}$
Silicon dioxide	26.0	$mg \cdot L^{-1}$
Sodium	1,840	$mg \cdot L^{-1}$
Strontium	4.24	$mg \cdot L^{-1}$
Sulfate	2,680	$mg \cdot L^{-1}$
TOC	12.38	$mg \cdot L^{-1}$

Table S2. Summary of one-way ANOVA results for the effects of pH, ionic strength, co-existing ions, and HA on NTMP degradation.

**pH (Group 1)**	**pH (Group 2)**	**Tukey HSD *p*-value**
pH 3.5	pH 7	0.8595
pH 3.5	pH 9	0.0006
pH 7	pH 9	0.0003
**Ionic strength (Group 1)**	**Ionic strength (Group 2)**	
0.12 M NaCl	0.6 M NaCl	0.9969
0.12 M NaCl	1.4 M NaCl	0.6558
0.12 M NaCl	Control	0.4934
0.6 M NaCl	1.4 M NaCl	0.7658
0.6 M NaCl	Control	0.3943
1.4 M NaCl	Control	0.1086
**Co-existing ions (Group 1)**	**Co-existing ions (Group 2)**	
Ca^{2+}	Control	0.0005
Ca^{2+}	HCO_3^-	0.0298
Ca^{2+}	K^+	0.0049
Ca^{2+}	Mg^{2+}	0.9877
Ca^{2+}	NO_3^-	0.0013
Ca^{2+}	SO_4^{2-}	0.0327
Control	HCO_3^-	0.3225
Control	K^+	0.8526
Control	Mg^{2+}	0.0019
Control	NO_3^-	0.9978
Control	SO_4^{2-}	0.3007
HCO_3^-	K^+	0.9473
HCO_3^-	Mg^{2+}	0.1088
HCO_3^-	NO_3^-	0.606
HCO_3^-	SO_4^{2-}	1
K^+	Mg^{2+}	0.0186
K^+	NO_3^-	0.9871
K^+	SO_4^{2-}	0.9345
Mg^{2+}	NO_3^-	0.0048
Mg^{2+}	SO_4^{2-}	0.1184
NO_3^-	SO_4^{2-}	0.5766
**HA (Group 1)**	**HA (Group 2)**	
2.3 mg-C·L^{-1} HA	4.6 mg-C·L^{-1} HA	0.3486
2.3 mg-C·L^{-1} HA	Control	0.1428
4.6 mg-C·L^{-1} HA	Control	0.0221

Table S3. Binding energy and full width at half-maximum (FWHM) of Fe(2p) in biotite before (0 h) and after (24 h) oxidation characterized by XPS.

	Name	*Peak BE*	*FWHM eV*	*Area (P) CPS.eV*	*Atomic %*
**0 h**	Fe^{2+} $2p_{3/2}$	709.18	2.09	4593.29	39.73
	Fe^{2+} $2p_{1/2}$	722.8	2.09	2582.33	
	Fe^{3+} $2p_{3/2}$	711.16	2.88	6954.81	60.27
	Fe^{3+} $2p_{1/2}$	724.84	2.88	3227.6	
	Fe^{2+} $2p_{3/2}$ satellite peak	714.46	3.85	5050.52	/
	Fe^{3+} $2p_{3/2}$ satellite peak	719.28	3.85	1786.19	
	Fe^{2+} $2p_{1/2}$ satellite peak	728.52	3.85	2525.32	
	Fe^{3+} $2p_{1/2}$ satellite peak	733.09	3.85	1114.81	

	Name	*Peak BE*	*FWHM eV*	*Area (P) CPS.eV*	*Atomic %*
**24 h**	Fe^{2+} $2p_{3/2}$	709.52	2.08	1587.21	24.9
	Fe^{2+} $2p_{1/2}$	723.05	2.08	872.83	
	Fe^{3+} $2p_{3/2}$	711.66	3.33	4776.42	75.1
	Fe^{3+} $2p_{1/2}$	725.13	3.33	2350.25	
	Fe^{2+} $2p_{3/2}$ satellite peak	714.88	3.94	2475.06	/
	Fe^{3+} $2p_{3/2}$ satellite peak	718.75	4.81	1206.45	
	Fe^{2+} $2p_{1/2}$ satellite peak	728.81	4.29	1495.36	
	Fe^{3+} $2p_{1/2}$ satellite peak	733.21	4.81	695.67	

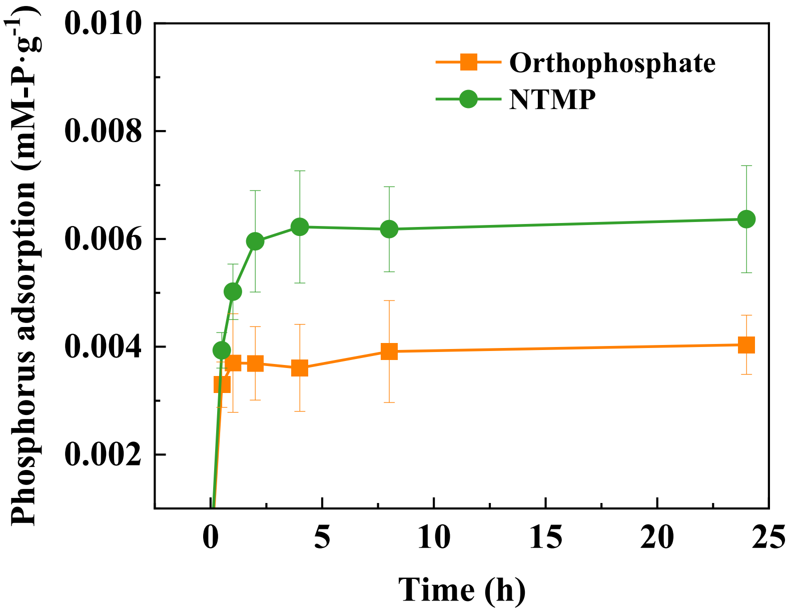

Fig. S1 Adsorption kinetics of orthophosphate and NTMP by biotite (1 g·L^{-1}) in 10 mM NaCl at pH 7. The initial concentrations of orthophosphate and NTMP were both 0.1 mM, each tested in separate experiments.

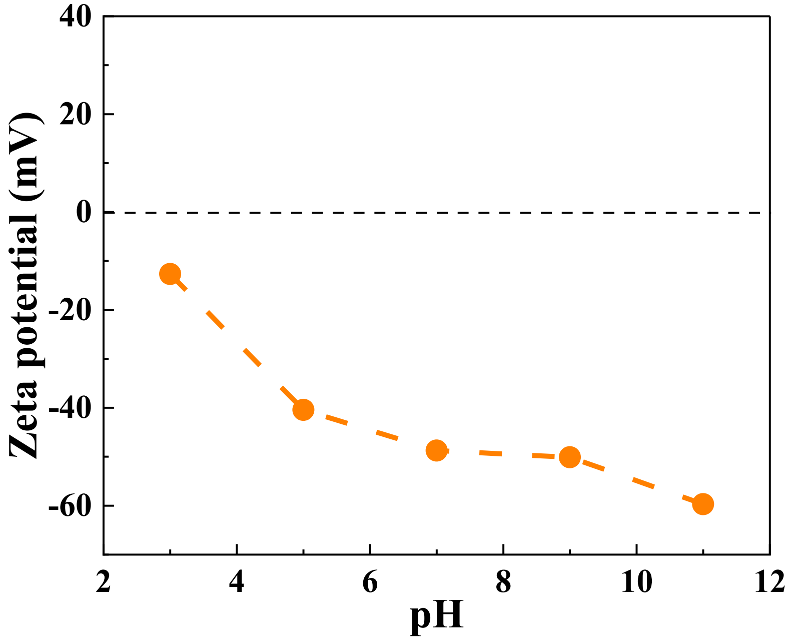

Fig. S2 Zeta potential of biotite particles at varied pH in 10 mM NaCl.

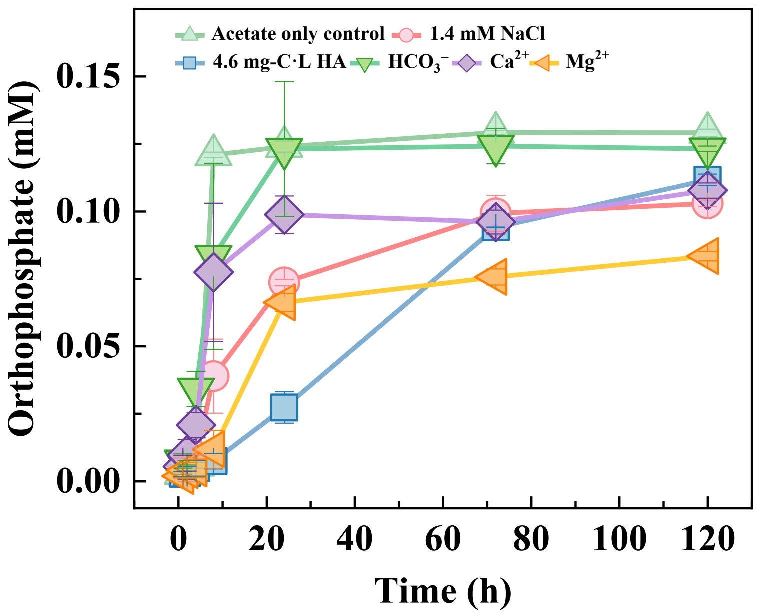

Fig. S3 Orthophosphate release from degradation of 0.1 mM NTMP in the presence of acetate with various inhibitors (i.e., 1.4 M NaCl, 4.6 mg-C·L^{-1}, 50 mM HCO_3^-, 70 mM Ca^{2+}, and 300 mM Mg^{2+}) by oxidation of 1g·L^{-1} biotite in 10 mM NaCl solution at pH of 7.

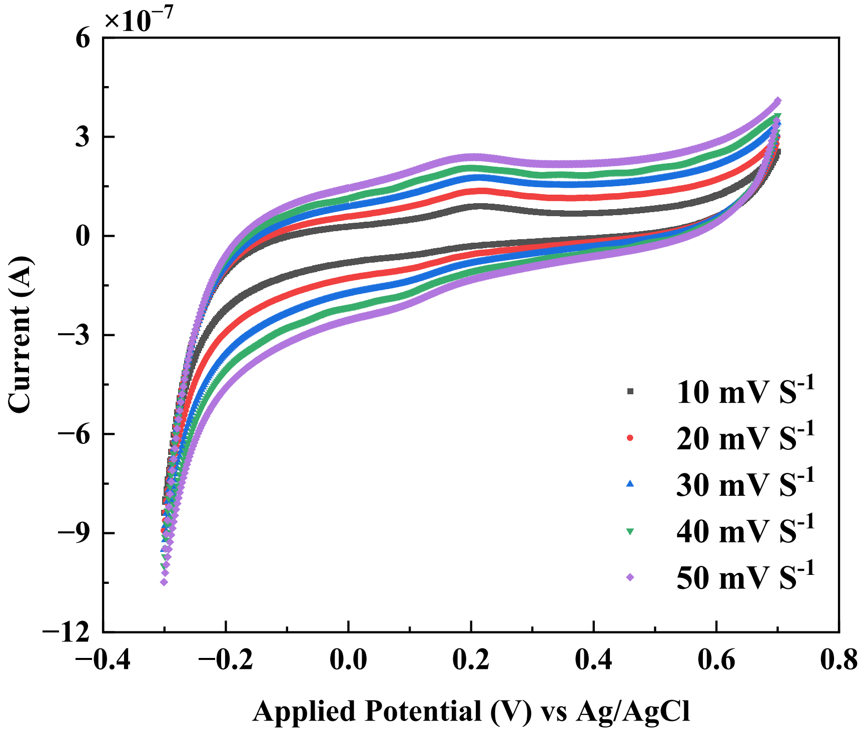

Fig. S4 Scan-rate-dependent cyclic voltammograms of biotite-graphite electrode with applied potential swept from −0.3 V to 0.7 V. Ag/AgCl was used as the reference electrode.
